# The Contribution of the Locus Ceruleus–Norepinephrine System to the Coupling between Pupil-Linked Arousal and Cortical State

**DOI:** 10.1523/JNEUROSCI.0898-25.2025

**Published:** 2025-12-02

**Authors:** Evan Weiss, Yuxiang Liu, Qi Wang

**Affiliations:** Department of Biomedical Engineering, Columbia University, New York, New York 10027

**Keywords:** adrenergic receptors, EEG, locus ceruleus, neural oscillations, norepinephrine, pupil-linked arousal

## Abstract

Understanding how pupil-linked arousal couples with cortical state is crucial for uncovering the neural mechanisms underlying brain state-dependent cognitive and sensory processing. Pupil size fluctuations reflect rapid changes of the pupil-linked arousal system, indexing brain states as well as the activity of neuromodulatory systems, including the locus ceruleus–norepinephrine (LC–NE) system. We investigated the relationship among phasic pupil dilation, cortical state, and neuromodulation by combining optogenetic LC stimulation with electroencephalogram (EEG) recordings and pupillometry in awake mice of both sexes. A comparison between EEG signals during spontaneous phasic pupil dilation and those during phasic pupil dilation evoked by LC stimulation revealed distinct cortical states. Using machine learning techniques, we trained a convolutional neural network classifier to distinguish between types of pupil dilation based on the power dynamics of individual EEG frequency bands. The results confirmed that all EEG bands, but most significantly gamma, differ markedly between spontaneous phasic arousal and LC stimulation-evoked arousal. Moreover, pharmacological manipulations to either block α- or β-adrenergic receptors or agonize α-2–adrenergic receptors were employed to explore how adrenergic receptors could influence the coupling between phasic pupil dilation and cortical state. With each manipulation uniquely modulating EEG power and pupil size, our results highlight the differentiated role of adrenergic receptors in moderating the coupling between pupil-linked arousal and cortical state. This study provides new insights into the complex relationship between pupil-linked arousal and cortical arousal state, underscoring the significant role of the LC–NE system in influencing these arousal states.

## Significance Statement

This study reveals the role of the locus ceruleus (LC) in pupil-linked arousal coupling with cortical arousal state by uncovering the different relationships between LC stimulation-evoked versus spontaneous phasic pupil dilations and cortical electroencephalogram. By integrating machine learning and noradrenergic pharmacological manipulation, our findings highlight the distinct cortical state associated with spontaneous phasic arousal and LC stimulation-evoked arousal, as well as the crucial role of different subtypes of adrenergic receptors in moderating the coupling between pupil size and cortical state.

## Introduction

The brain operates in various arousal states in our daily lives, which are regulated by central arousal systems and characterized by several physiological signals (Gilbert and Sigman, 2007; Niell and Stryker, 2010; Lee and Dan, 2012; Bennett et al., 2014; Poulet and Crochet, 2018). Historically, oscillation patterns in electroencephalogram (EEG) have been used to characterize cortical state, which reflects a mode of population-level cortical neuronal activity (Buzsáki and Draguhn, 2004; Jacobs et al., 2020). The distribution of power across different EEG frequency bands (i.e., delta, theta, alpha, beta, gamma, or coarsely, low vs high frequencies) has shown to correlate with transitions between wakefulness and sleep and therefore is widely believed to reflect the brain's arousal level (Steriade, 1996). Although other physiological signals, such as heart rate variability, have been shown to index arousal state (Mathias and Stanford, 2003; Azarbarzin et al., 2014; Mather and Thayer, 2018; Wang et al., 2018; Liu et al., 2021), recent work has provided experimental evidence that fluctuations in the pupil size are also an indicator of the activity of a central arousal system (i.e., pupil-linked arousal; Nassar et al., 2012; Murphy et al., 2014; Reimer et al., 2014; Ebitz and Platt, 2015; McGinley et al., 2015; Vinck et al., 2015; Urai et al., 2017; de Gee et al., 2020). This noninvasive measure of central arousal offers new insight into the effect of brain state on sensory and cognitive processing (Hess and Polt, 1960; Reimer et al., 2014, 2016; Oliva and Anikin, 2018), as changes in the pupil size provide a quantitative metric about cognitive functions, making it a valuable tool for investigating how these changes influence neural coding and information processing (Gilzenrat et al., 2010; van Kempen et al., 2019; Zhao et al., 2019; Joshi and Gold, 2020; Schriver et al., 2020; Lapborisuth et al., 2022; Strauch et al., 2022).

**Figure 1.**
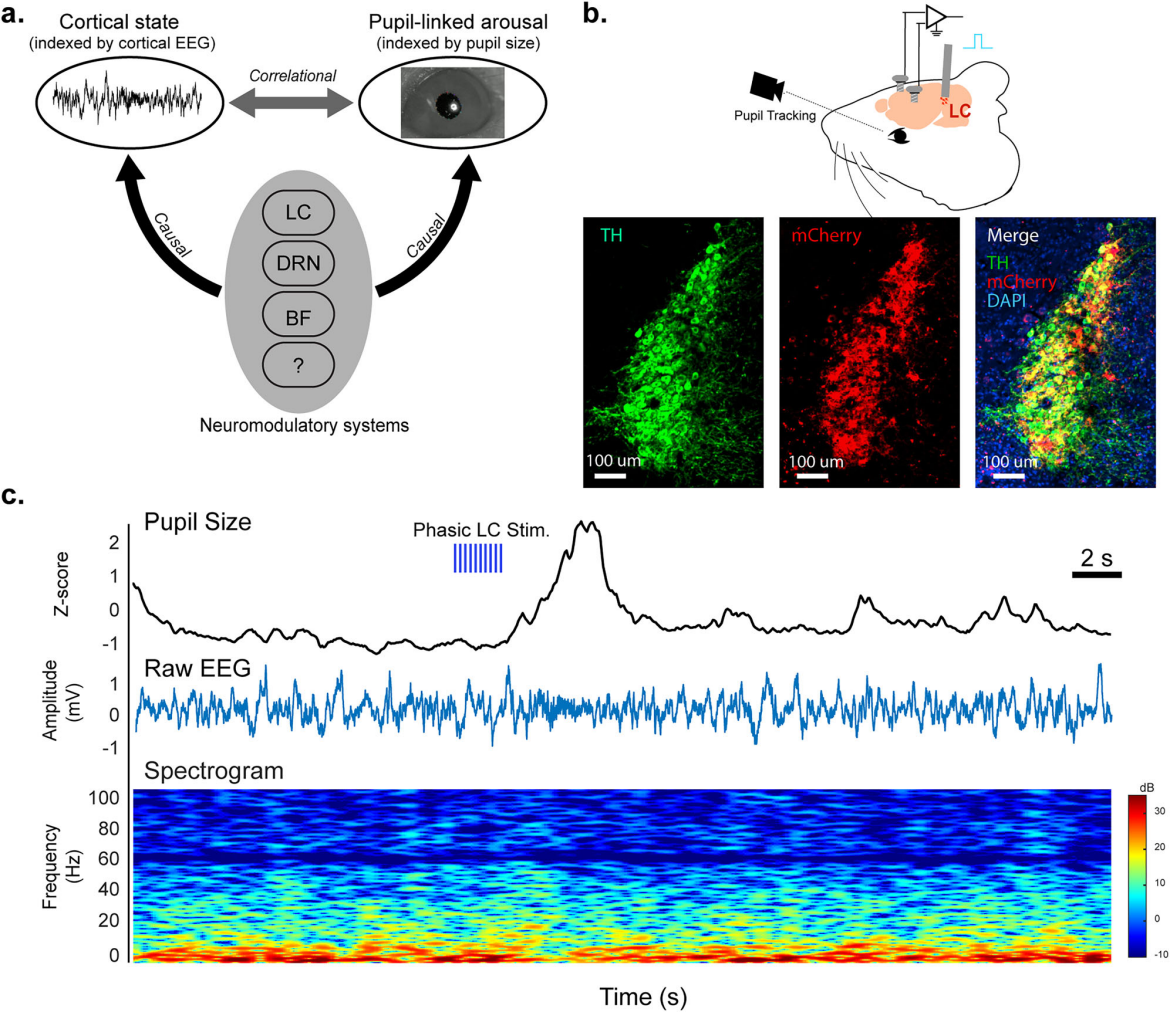
LC stimulation evokes pupil dilations and EEG spectral changes. ***a***, Diagram of multiple neuromodulatory systems moderating the coupling between pupil-linked arousal and cortical state. ***b***, Diagram of simultaneous pupil and EEG recording during optogenetic LC stimulation (top) and histological confirmation of the expression of ChR2 in LC neurons (bottom). ***c***, Visualization of a typical stimulation trial. Phasic pupil dilation evoked by phasic LC stimulation (top), raw EEG signal during stimulation period (middle), and an EEG spectrogram during same time period (bottom).

Several lines of evidence have suggested that multiple neuromodulatory systems regulate pupil-linked arousal. Work by our group and others established the causal link between locus ceruleus (LC) activation and pupil dilation in both rodents and nonhuman primates (Joshi et al., 2016; Liu et al., 2017). In these studies, a burst of microstimulation of the LC mimicking phasic firing of LC neurons evoked phasic pupil dilations. It has also been demonstrated that the activation of the dorsal raphe nucleus altered the pupil size (Cazettes et al., 2021). Additionally, Reimer et al. (2016) showed strong correlations between the pupil size and the activity of both noradrenergic and cholinergic axons in the cortex, consistent with a recent result where the extracellular acetylcholine level in the prefrontal cortex was shown to covary with the pupil size (Liu et al., 2025). It is important to note that the activation of these neuromodulatory nuclei also shift the power distribution across different frequency bands (Weiss et al., 2023). For instance, Liu et al. (2017) have demonstrated that phasic microstimulation of the LC of rats desynchronized cortical EEG activity by shifting EEG power from low-frequency bands (1–10 Hz) to high-frequency bands (10–100 Hz). Moreover, studies employing pharmacological manipulation of adrenergic receptors (α-1, α-2, and β) have showcased broad cortical state changes and behavioral consequences (Roubicek, 1976; Pastel and Fernstrom, 1984; Riekkinen et al., 1990; Sainsbury and Partlo, 1993; Villain et al., 2016; Fitzgerald, 2021). Similar to LC stimulation, electrical microstimulation of the nucleus basalis, the cholinergic nucleus of the basal forebrain, increased the ratio of power in high-frequency bands relative to low-frequency bands of cortical LFP (Goard and Dan, 2009). As studies have demonstrated that spontaneous fluctuations in pupil-linked arousal are correlated with changes in cortical activity (Breeden et al., 2017), these neuromodulatory systems likely play a critical role in facilitating the coupling between pupil-linked arousal and cortical state (Fig. 1*a*). However, little is known about the extent to which these neuromodulators contribute to the coupling.

In this study, we aim to distinguish the role of the LC–norepinephrine (NE) system from the other neuromodulatory systems in moderating the coupling between phasic pupil-linked arousal and cortical state. By quantifying the difference between EEG activity during spontaneous phasic pupil dilation periods and LC stimulation-evoked phasic pupil dilation periods, we found distinct EEG dynamics across the six canonical frequency bands during the two types of phasic pupil dilations. To further quantify differences, we developed a convolutional neural network (CNN) classifier to decode the type of pupil dilation from EEG power across each individual frequency band, confirming that spontaneous and LC stimulation-evoked phasic arousal exert distinct effects on EEG power, particularly in gamma frequencies. Furthermore, we pharmacologically manipulated adrenergic receptors using propranolol, phentolamine, and clonidine. Each drug altered underlying spontaneous pupil dynamics as well as EEG power across frequency bands. Taken together, these results provide new insights into the complex coupling between pupil-linked arousal and cortical state, highlighting the unique influence of the LC–NE system in the coupling.

## Materials and Methods

All experimental procedures involving animals were approved by the Columbia University Institutional Animal Care and Use Committee (IACUC) and were conducted in compliance with NIH guidelines. Adult Dbh-Cre mice (RRID: IMSR_JAX:033951, Jackson Laboratory) of both sexes (11 mice, 5 females), aged 3–7 months, were used in the experiments. All mice were kept under a 12 h light/dark cycle. All recordings were conducted in awake, head-fixed animals that were not engaged in behavioral tasks.

### Surgical procedures

In aseptic setup, mice were anesthetized with isoflurane in oxygen (5% induction, 2% maintenance) and fixed in a stereotaxic frame. Body temperature was maintained at 36.6°C using a feedback-controlled heating pad (FHC). Following the removal of fur from the scalp and cleaning of the surgical site, lidocaine (0.1 ml, SC) was injected to the scalp to provide local anesthesia. Buprenorphine (0.05 mg/kg, sc) was then administered to ensure analgesics were on board throughout the surgery.

For adeno-associated viral (AAV) vector injections, a burr hole was drilled above the left LC. Pulled capillary glass micropipettes (Drummond Scientific) were back-filled with AAV solution and injected into the target brain regions at 0.7 nl/s using a precision injection system (Nanoliter 2020, World Precision Instruments). The pipette was left in place for at least 10 min between injections and slowly withdrawn. To optogenetically activate the LC, pAAV-EF1a-double-floxed-hChR2-(H134R)-mCherry-WPRE-HGHpA (Addgene #20297-AAV9, 250 nl) was injected into the LC (AP, −5.3 mm; ML, 0.85 mm; DV, −3 mm). Immunohistology staining for tyrosine hydroxylase (TH) confirmed the expression of mCherry-tagged ChR2 in TH-positive neurons in the LC (Fig. 1*b*). To measure NE dynamics during LC stimulation, AAV encoding GRAB_NE_ (AAV9-hSyn-NE2 h) was injected into the prefrontal cortex (AP, +2.0 mm; ML, 1.0 mm; DV, −1.3 mm). After injection, an optical fiber (200 µm diameter and NA, 0.39) was implanted with the tip of the fiber placed ∼150 µm above injection site. C&B Metabond (Parkell) was used to build a headcap to bond the ferrule and the head bar.

For EEG electrode implantation, three burr holes were drilled above the prefrontal cortex (bilaterally, ML, ±1.0 mm; AP, +2.0 mm) and occipital lobe (ML, −1.0 mm; AP, −4.0 mm), with saline applied to each craniotomy to prevent drying out of brain surface. Three EEG recording screw electrodes (NeuroTek-IT) were then threaded into the skull to gently touch the brain surface. The wire of the EEG screw electrodes was connected to a custom-made EEG head stage with conductive epoxy (MG Chemicals 8331S-15G). The head stage was then bonded to the headcap with light-curing dental cement (Prime Dental Manufacturing). At the conclusion of the surgery, Baytril (5 mg/kg) and ketoprofen (5 mg/kg) were administered. Four additional doses of Baytril and two additional doses of ketoprofen were provided every 24 h after the surgery day. All recordings were performed at least 3 weeks after surgery to allow sufficient time for viral expression.

### Pharmacological manipulation

The pharmacological manipulation of the LC–NE system was done through administration of three FDA-approved adrenergic drugs: propranolol hydrochloride (Spectrum Laboratory Products PR 140), a nonselective beta blocker (Villain et al., 2016), phentolamine hydrochloride (Sigma-Aldrich P7547), an α-adrenoceptor antagonist (Starke et al., 1971; Cazala, 1980), and clonidine hydrochloride (Spectrum Laboratory Products CL118), an α-2–adrenergic agonist (Delbarre and Schmitt, 1971; Kleinlogel et al., 1975; Nelson et al., 1985). Each drug was dissolved in saline and sterile filtered before use and was administered intraperitoneally ∼15–30 min before sessions. Dosage in milligrams per kilogram was calculated based on experimental animals' daily weight. Propranolol was dosed at 1 mg/kg, phentolamine at 5 mg/kg, and clonidine at 1 mg/kg. Saline control sessions were randomly interleaved daily during experimental recording periods.

### Histology

At the end of the study, mice were transcardially perfused with PBS followed immediately by ice-cold 4% paraformaldehyde. The brain was removed carefully and postfixed overnight at 4°C in 4% paraformaldehyde and then cryopreserved in a 30% sucrose (wt/vol) in PBS solution for 3 d at 4°C. Brains were embedded in optimum cutting temperature compound, and 25 µm coronal slices were sectioned using a cryostat. Brain slices were washed four times in PBS and then incubated in 10% normal goat serum contained with 0.5% Triton X-100 in PBS for 2 h. This was followed by primary antibody incubation overnight at room temperature using a chicken anti-TH (1:500) primary antibodies. On the next day, slices were washed three times in PBS + Tween 20 (0.0005%) solution followed by secondary antibody incubation for 2 h at room temperature using an Alexa Fluor 488-conjugated goat anti-chicken (1:800). The slices were then washed three times in PBS + Tween 20 solution and one time with PBS only followed by coverslipping using Fluoromount-G medium with DAPI. Selected example slices were imaged using 10× under a confocal microscope (Nikon Ti2) with a spinning disk (Yokogawa CSU-W1).

### Optogenetic stimulation and EEG recording

To photo stimulate LC neurons expressing ChR2, blue light generated by a LED module was delivered through the implanted optical fiber (*λ* = 473 nm, PlexBright, Plexon). During recordings, the mouse sat in a 3D-printed head–fixation platform. Every 30 s, an optogenetic stimulation with either 5 or 10 Hz (randomly interleaved, 10 ms pulse duration) was delivered for a 2 s duration. EEG signals were digitalized by a 16-channel recording head stage (Model #C3335, Intan Technologies) and subsequently recorded by a RHD USB interface board (Model #C3100, Intan Technologies). A Bpod State Machine (Sanworks) was used with custom Python experimental scripts to synchronize optogenetic stimulation events, EEG recording, and pupil camera acquisition.

### Fiber photometry recording and preprocessing

A two-channel fiber photometry system (Doric Lenses) was used to capture fluorescence signals from the GRAB_NE_ sensors. Each sensor recording utilized LED-generated excitation light at 465 nm (CLED_465, Doric Lenses), which was direct through a MiniCube [iFMC4_AE (405)E(460–490)_F(500–550)_S, Doric Lenses]. The MiniCube's integrated PMT detector measured the resulting emission fluorescence from the GRAB_NE_ sensors. Recordings were performed using “Lock-in” mode via the Doric Neuroscience Studio software (V5.4.1.12), with the four excitation light sources modulated at distinct frequencies (208.62, 572.21, 333.79, and 470.88 Hz) to prevent interference from ambient light and eliminate cross-contamination between excitation channels. The Doric fiber photometry console demodulated the signal, applied a 25 Hz low-pass filter, and digitized it at 12 kHz using 16 bit ADC. TTL signals from an xPC target real-time system (MathWorks) enabled synchronization between the photometry system and stimulation hardware. The Doric Neuroscience Studio software downsampled all photometry data to 120 Hz before storing it for subsequent analysis. To examine NE dynamics specifically during LC stimulation intervals, the fluorescence signal underwent high-pass filtering at 0.1 Hz to eliminate low-frequency oscillations before calculating NE dynamics within the stimulation timeframe.

### Pupillometry and pupil size extraction

Pupil recordings were obtained using a custom pupillometry system under constant luminance in a quite experimental room (Liu et al., 2025). The camera was triggered by 10 Hz TTLs from the Bpod State Machine. Pupil images were streamed to a high-speed solid–state drive for offline analysis. For each video clip, a region of interest was manually selected initially. The DeepLabCut toolbox was used to segment the pupil contour (Mathis et al., 2018). Training sets were created, consisting of 90 frames for video clips with the resolution of 1,280 × 1,080 pixels. Within each frame, 12 points around the pupil were manually labeled, and cropping parameters were adjusted to enhance training accuracy. The resnet_50 deep network was trained on each frame and employed for the analysis of video clips from all sessions. Circular regression was then applied to fit the automatically labeled points, enabling the computation of the pupil size based on the fitted contour. To ensure segmentation accuracy, ∼5% of segmented images were randomly selected and inspected. The pupil size during periods of blinks was estimated through interpolation, using pupil sizes just before and after blinks. Prior to further analysis, a fourth-order noncausal low-pass filter with a cutoff frequency of 3.5 Hz was applied to the pupil size data (Schriver et al., 2018). Additionally, periods of pupil activity during rapid eye movements were excluded from the analysis.

### Data analysis

All data analyses were first conducted on individual sessions. Grand averages and standard errors of means were then calculated across sessions or animal subjects for analysis and visualization.

### Spontaneous pupil dilation detection

The pupil size of each session was first *z*-scored and then subsequently upsampled from 10 to 100 Hz using spline interpolation. Upsampling was necessary to match our EEG power time series to our pupil data for the machine learning analyses. Next, a high-pass noncausal Butterworth filter was used to remove low-frequency drifts from the pupil data with a cutoff frequency of 0.1 Hz. To detect spontaneous pupil dilations, we used the following protocol and criteria for consistency across sessions and animals: the pupil size time series was iteratively segmented into 10 s windows, sliding through the entire time series for each session in 0.01 s increments. For each window, a baseline stability period was confirmed by checking whether the first 2 s remained continuously below a *z*-score threshold of 0.5, ensuring the pupil size was stable before detecting a peak. If baseline criterion was met, the algorithm searched for peaks within the same 10 s window where the *z*-score exceeded 0.5 (i.e., 0.5 standard deviation). This *z*-score threshold yielded similar amplitudes of spontaneous phasic pupil dilations and LC stimulation–evoked pupil dilations (Fig. 3*a*). We also tested other *z*-score thresholds (0.65 and 1), which produced results similar to those obtained with the threshold of 0.5 (Fig. S4). The first detected peak was identified as the max of the dilation event. After the peak, the algorithm checked whether the pupil returned to baseline (below the baseline *z*-score threshold) within 6 s, ensuring short, transient dilations were captured. Each dilation was bounded by the period preceding the first peak and the interval following it. Spontaneous dilations events were aligned by their peak, providing a common reference point and a central point treated as time 0 across trials (Fig. 3*a*). Once a dilation was identified, the algorithm will search for the next spontaneous dilation from the offset of the current dilation, and this process was repeated through the recording of the entire session.

For LC stimulation-evoked dilations, the alignment windows were also aligned by the peak of the pupil dilation, identical to the spontaneous method described above. Comparing this method with alignment of LC stimulation-evoked dilations to stimulation onset yielded similar pupil dilation in peak dilation and baseline size (Fig. S2*a*). This approach ensured identically sized and aligned temporal windows, allowing for a fair comparison of the time course and magnitude of the pupil responses across spontaneous and LC stimulation-evoked conditions. Pupil baseline for both dilation types was defined as the average pupil size from −6 to −4 s prior to the peak of dilations, while pupil dilation amplitude was defined as the average pupil size from −2.5 to 2.5 s around dilation peaks minus the pupil baseline.

### EEG processing and analysis

The recorded EEG was first filtered with a noncausal sixth order low-pass Butterworth filter with a cutoff frequency of 100 Hz to remove high-frequency noise. To align the 100 Hz pupil time series with the 1,000 Hz original sampling frequency of the EEG data, we used a sliding window technique. This downsampled the EEG data to match the time resolution of the pupil data, ensuring both signals were temporally aligned and suitable for analysis. The moving window analysis was implemented by segmenting the EEG data sampled at 1,000 Hz into 1 s windows, each containing 1,000 samples. To ensure smooth temporal coverage, the step size was set to 10 samples (10 ms), ensuring the resulting EEG power series had the same 100 Hz as the pupil data. For each sliding window, we computed its power spectral density (PSD) using the fast Fourier transform of the EEG signal within that window (Delis et al., 2018). To evaluate the power within each frequency band, we computed the area under the curve (AUC) of the PSD for specific frequency bands: delta (1–4 Hz), theta (4–8 Hz), alpha (8–12 Hz), beta (12–30 Hz), low gamma (30–55 Hz), and high gamma (65–100 Hz). The AUC for each frequency band was calculated using MATLAB's *trapz* function, approximating the integral of the PSD curve, yielding average power within that frequency range. This process was repeated every 10 ms across the entire dilation period, producing a PSD time series that matched the resolution of the pupil data (Fig. 4*a*).

For aperiodic analysis, the raw EEG signal following LC stimulation (Fig. S1) or encompassing the 12 s period centered around the peak pupil dilation (Fig. S3) was parameterized with FOOOF in Python (Donoghue et al., 2020). Across all conditions, the same FOOOF parameters were a frequency range of 2–55 Hz (due to notch filtering at 60 Hz), a peak_width_limit = [2,10], aperiodic_mode = “knee”, min_peak_height = 0.1, and a max_n_peaks = 3. Results are represented as session averages.

### Machine learning analyses

#### CNN classifier analysis

To classify the types of phasic pupil dilation from EEG band power, a CNN was applied. For each frequency band, the PSD time series data, temporally centered around peak pupil dilation, was processed. This included the construction of a dataset which included all trials from all experimental sessions across all animals. A separate neural network model, with the architecture detailed below, was individually trained and evaluated for each frequency band. Model performance was assessed using a fivefold cross-validation scheme. Each model was trained using the Adam optimizer with a minibatch size of 32 sequences. Training was conducted for a maximum of 100 epochs. An early stopping criterion, monitoring the validation accuracy, was employed to prevent overfitting and ensure that the model weights from the epoch yielding the highest validation accuracy were retained. Our framework was implemented in Python using the TensorFlow library with the Keras API. All training and validation were executed on an NVIDIA A100 GPU.

Our CNN employed ReLU activation, adjusting a feature space for each layer:

$$x_{i}=\mathrm{ReLU}(\omega_{i}\;*\;x+b_{i}),$$

where $\omega_{i}$ are the filters, * denotes convolution operation, $b_{i}$ are the biases, and $x_{i}$ is the output feature map after activation.

The network's output followed a single-unit dense layer with sigmoid activation function to output a probability indicating the class label, using the following activation:

$$y=\frac{1}{1+e^{-z}},$$

where *z* is the input into the sigmoid function and y is the output probability.

#### Support vector machine (SVM) analysis

A radial basis function (RBF) kernel SVM was implemented to capture potential nonlinear relationships in the data. Hyperparameter optimization was performed using grid search cross-validation, tuning the regularization parameter C and the RBF kernel coefficient γ. The decision function for the RBF-SVM is given as follows:

$$f(x)=\underset{i}{\sum }\;(\alpha_{i}y_{i}(x_{i}\cdot x))+b,$$

where $K(x_{i},\;x)=e^{\left(-\gamma {\left|\left|x_{i}-x\right|\right|}^{2}\right)}$ is the RBF kernel, $\alpha_{i}$ are the Lagrange multipliers, and b is the bias term.

#### Random forest classifier analysis

An ensemble of 500 decision tress was constructed with maximum depth set to prevent overfitting while maintaining model complexity. Key hyperparameters included minimum samples per split (between 2 and 20), minimum samples per leaf (1–10), and maximum features considered at each split.

#### Linear discriminant analysis (LDA) analysis

LDA was employed to find the linear combination of features that maximizes the ratio between-class to within-class scatter. The discriminant function seeks to maximize the following:

$$J(w)=\frac{w^{T}S_{\beta }w}{w^{T}S_{v}w},$$

where $S_{\beta }$ represents the between-class scatter matrix and $S_{v}$ represents the within-class scatter matrix.

#### Elastic Net regularization analysis

To address potential multicollinearity among frequency bands and perform implicit feature selection, we implemented Elastic Net logistic regression, which combines L1 and L2 penalties as follows:

$$\min\_w\left(\frac{1}{2n}\right)||Xw-y|{|}^{2}+\lambda \left(\alpha ||w|{|}_{1}+\frac{\left(1-\alpha \right)}{2}||w|{|}_{2}^{2}\right)$$

#### Multilayer perceptron (MLP) analysis

A fully connected feedforward neural network was implemented with two hidden layers (128 and 64 neurons, respectively) using ReLU activation functions. The network employed dropout regularization between layers to prevent overfitting. Training utilized the Adam optimizer with batch normalization applied after each hidden layer to accelerate convergence and improve generalization.

All models underwent stratified fivefold cross-validation to ensure balanced class representation across folds. The same data splits were maintained across all algorithms to enable direct performance comparison. Implementation utilized scikit-learn (version 1.3.0) for all traditional machine learning algorithms. All models were trained on the same preprocessed feature sets as the CNN to ensure consistency in comparative analysis.

### Statistics

All statistical tests were two-sided. If the samples were normally distributed, a paired or unpaired *t* test was used. For comparisons involving more than two groups, a one-way ANOVA was conducted, followed by a post hoc Tukey’s test to account for multiple comparisons. If the data did not meet the assumptions of normality, a Mann–Whitney *U* test was employed as a nonparametric alternative for comparisons between two groups, and a Wilcoxon signed rank test was used for paired samples.

## Results

### LC stimulation evokes pupil dilations and EEG spectral changes

While previous behavioral studies have quantified the LC's ability to modulate arousal, a clear investigation into its role in moderating the relationship between pupil-linked arousal and cortical state is missing. To address this question, we employed optogenetic stimulation to selectively activate LC neurons while simultaneously recording pupil and EEG activity (Fig. 1*b*). In Dbh-Cre mice, we injected a pAAV-EF1a-double-floxed-hChR2-(H134R)-mCherry-WPRE-HGHpA viral vector to drive channelrhodopsin (ChR2) expression specifically in LC neurons, activating them by delivering blue light through an optical fiber implanted above the LC. While optically stimulating the LC, we simultaneously recorded the animal's pupil size and EEG signals via the implanted skull electrodes (Fig. 1*b*). Postmortem immunohistochemistry staining verified the ChR2 expression in the LC, showing robust overlap between mCherry, a tag of the AAV, and TH signals, a hallmark of LC neurons (Fig. 1*b*). We also observed phasic pupil dilations and changes in EEG spectrum following optogenetic LC stimulation (Fig. 1*c*). Although these changes are believed to primarily result from activation of the LC–NE system, indirect activation of other neuromodulatory systems that receive projections from the LC might also contribute to the changes (see Discussion).

**Figure 2.**
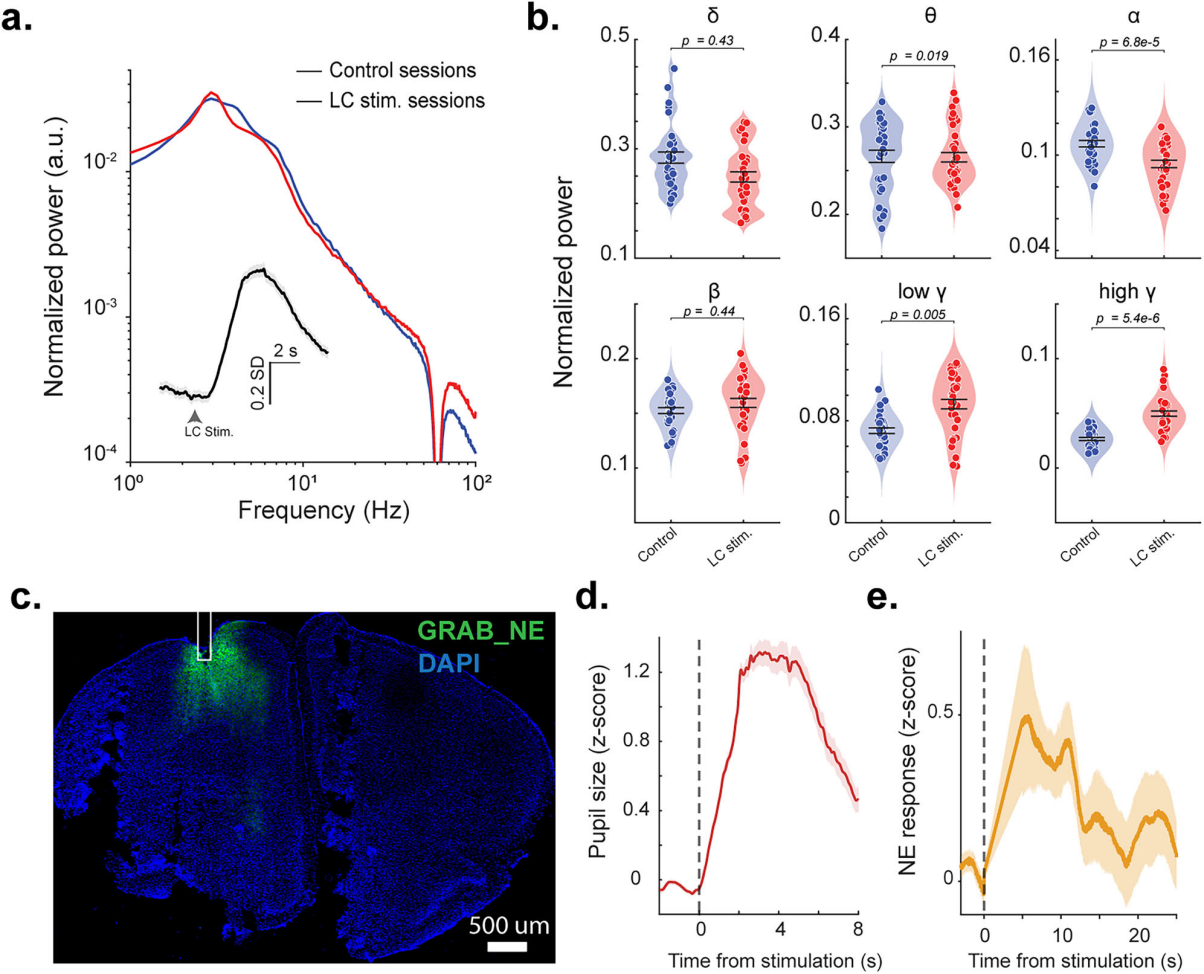
EEG spectrum of LC stimulation and control sessions. ***a***, Normalized PSD spectrum between spontaneous and LC stimulation sessions. Inset, the pupil size aligned to LC stimulation onset. ***b***, Normalized power over each frequency band (δ (1–4 Hz), θ (4–8 Hz), α (8–12 Hz), β (12–30 Hz), low γ (30–55 Hz), and high γ (65–100 Hz) for LC stimulation sessions and control sessions. ***c***, Histological confirmation of expression of GRAB_NE_ in the prefrontal cortex around where EEG electrodes were implanted. ***d***, The pupil size locked to LC stimulation onset in GRAB_NE_ animals (*n* = 2). ***e***, NE dynamics locked to LC stimulation onset of the LC, returning to baseline levels in ∼20 s.

To further assess the distinct effects LC stimulation has on cortical EEG, we compared the EEG spectrum between LC stimulation sessions and nonstimulation control sessions to quantify the extent to which elevated LC activity affects cortical arousal state. In each mouse, we recorded multiple spontaneous (i.e., no LC stimulation) sessions as well as LC stimulation sessions and calculated normalized PSD, which allows for between-session comparisons (Fig. 2*a*). Consistent with previous results showing that LC-driven shifts in cortical arousal, here we observed decreases in low-frequency power and increases in high-frequency power in LC stimulation sessions compared with control sessions (randomly sampled 2 s periods of activity; Berridge and Foote, 1991; Bouret and Sara, 2005). Further quantifying EEG power band by band confirmed significant increases in normalized power within the low gamma (*p* = 0.005, Mann–Whitney *U* test; Fig. 2*b*) and high gamma bands(*p* = 5.4 × 10^−6^, Mann–Whitney *U* test; Fig. 2*b*), as well as a significant decrease in theta power (*p* = 0.019, Mann–Whitney *U* test; Fig. 2*b*) and alpha power (*p* = 6.8 × 10^−5^, Mann–Whitney *U* test; Fig. 2*b*), indicating that LC stimulation modulates cortical state. Comparison between raw EEG signals in sessions with and without LC stimulation exhibited similar trend but with a significance only in the high gamma band (Fig. S1*a,b*).

To further understand the difference in cortical state between spontaneous and LC stimulation conditions, we performed aperiodic analysis using FOOOF (see Materials and Methods). We found the spectra of EEG during LC stimulation had smaller aperiodic exponent (i.e., flatness of the spectrum in the log–log plot) than during control sessions (*p* = 2.51 × 10^−4^, Student's *t* test; Fig. S1*c*). Moreover, aperiodic offsets (i.e., intersect with *y*-axis) is also lower during LC stimulation sessions than during control sessions (*p* = 0.009, Student's *t* test; Fig. S1*d*). These results are consistent with studies showing a steeper aperiodic component is indicative of greater cortical arousal, desynchronization, as well as a more active cortical state (Lombardi et al., 2017; Lendner et al., 2020).

In order to confirm a reset in LC activity prior to the next LC stimulation trial, we performed fiber photometry experiments to record NE signals during phasic LC stimulation. Here we injected a genetically encoded NE fluorescent biosensor GRAB_NE_ AAV into the prefrontal cortex, while simultaneously optogenetically stimulating the LC in head-fixed Dbh-Cre mice. Histological verification confirmed the expressions of GRAB_NE_ in the prefrontal cortex, where our EEG screw electrode was placed (Fig. 2*c*). Using the same experimental paradigm as above, GRAB_NE_ animals experienced rapid pupil dilation in response to the photostimulation of the LC (Fig. 2*d*). As expected, NE levels in the prefrontal cortex rapidly increased following LC stimulation. Notably, NE signals returned to the baseline within 20 s, confirming that elevated arousal levels resulting from LC stimulation reset to baseline before future stimulation trials (Fig. 2*e*). We failed to observe poststimulation inhibition for the prefrontal NE levels, suggesting that optogenetic LC stimulation indeed increased overall LC firing rather than redistributing LC spikes over time.

**Figure 3.**
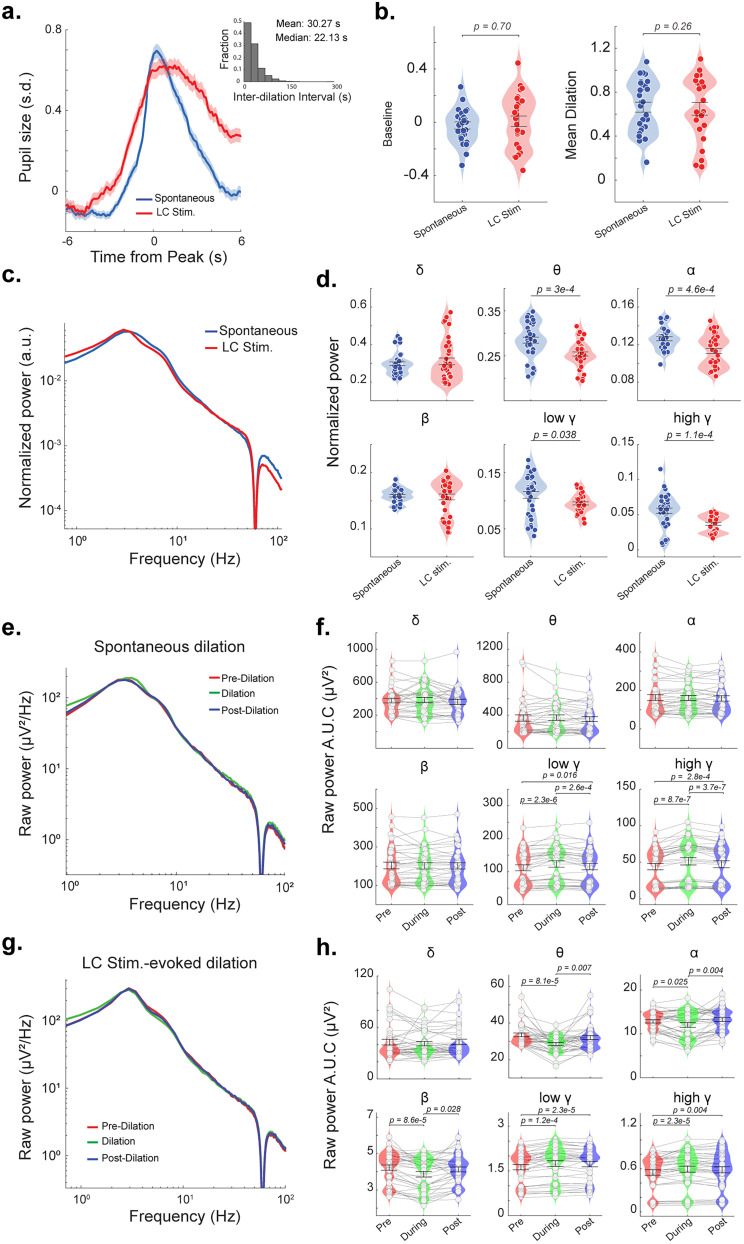
EEG spectrum around spontaneous and LC stimulation-evoked phasic pupil dilations. ***a***, The pupil size aligned to peak dilation (−6 to 6 s from peak) for spontaneous and LC stimulation-evoked pupil dilations. Inset, Histogram of interdilation intervals of spontaneous phasic dilations; mean time between dilations, 30.27 s; median, 22.13 s. ***b***, Quantification of pupil baseline (left) and mean dilation size (right) for spontaneous and LC stimulation-evoked phasic pupil dilations. ***c, d***, Normalized EEG power spectrum and power in each frequency band during spontaneous and LC stimulation-evoked pupil dilations. ***e, f***, Raw EEG PSD and power in each frequency band before, during, and after spontaneous phasic pupil dilations. ***g, h***, Raw EEG PSD and power in each frequency band before, during, and after LC stimulation-evoked phasic pupil dilations.

As previous work suggests that the activation of other neuromodulatory systems other than the LC–NE system can affect the pupil size, these neuromodulatory systems may also contribute to the coupling between pupil-linked arousal and cortical state. To better understand the role of the LC–NE system in moderating the coupling between pupil-linked arousal and cortical state, we compared cortical EEG signals during spontaneous phasic pupil dilation periods and LC stimulation-evoked pupil dilation periods. To this end, we first identified spontaneous phasic pupil dilations in control sessions (see Materials and Methods) to match the size of a typical LC-evoked pupil dilation using a detection threshold of 0.5 SD. On average, spontaneous phasic dilations occurred every 30.27 s, which is comparable to intervals of LC stimulations (Fig. 3*a* inset). However, unlike LC stimulation-evoked pupil dilations, the onset of spontaneous phasic pupil dilations is hard to measure. Therefore, we aligned these two types of dilations by their peaks (Fig. 3*a*). Notably, aligning LC stimulation-evoked pupil dilations by the onset of optogenetic stimulation did not significantly differ from these pupil dilations aligned by their peaks (Fig. S2). Both spontaneous phasic pupil dilations and LC stimulation evoked dilations had a nonsignificant difference in both baseline and dilation amplitude (*p* = 0.70 and *p* = 0.26, respectively, Student's *t* tests; Fig. 3*b*), confirming a fair comparison for both types of dilations.

We next looked to quantify different EEG power distributions during LC stimulation-evoked versus spontaneous pupil dilations. Computing the normalized EEG power spectrum during each type of pupil dilation periods, encompassing the 12 s period centered around the peak of the dilation, revealed a distinct difference in low- and high-frequency band power (Fig. 3*c*). Notably, EEG signals during LC stimulation-evoked pupil dilation periods had significantly lower power in the theta (*p* = 3 × 10^−4^, Student's *t* test; Fig. 3*d*), alpha (*p* = 4.6 × 10^−4^, Student's *t* test; Fig. 3*d*), low gamma (*p* = 0.038, Student's *t* test; Fig. 3*d*), and high gamma (*p* = 1.1 × 10^−4^, Student's *t* test; Fig. 3*d*) bands. Moreover, aperiodic analyses revealed that EEG spectra during LC stimulation-evoked dilations experienced a significantly lower aperiodic exponent (*p* = 0.0001, Student's *t* test; Fig. S3*a*,*b*) and a lower aperiodic offset (*p* = 0.001, Student's *t* test; Fig. S3*c*) than those during spontaneous dilations, further confirming the differences in cortical state between the two arousal periods.

After identifying different cortical states associated with spontaneous pupil dilations and LC stimulation-evoked pupil dilations, we next compared cortical states right before, during, and after each of the two types of pupil dilations to gauge the time course of the coupling between pupil-linked arousal and cortical state. To this end, we calculated EEG spectrum within three 5 s periods, i.e., [−7.5 −2.5], [−2.5 2.5], and [2.5 7.5] seconds relative to the peak of pupil dilations. During spontaneous pupil dilations, there was a significant increase in power in both the low and high gamma bands compared with predilation periods (*p* = 2.3 × 10^−6^ and *p* = 8.7 × 10^−7^, respectively, repeated-measure ANOVA with Tukey's post hoc test; Fig. 3*f*). On the contrary, in LC stimulation conditions, we observed significant changes in power in all bands except delta between the dilation periods and the predilation periods. Specifically, there was a decrease in power in the theta, alpha, and beta bands (*p* = 8.1 × 10^−5^, *p* = 0.025, and *p* = 8.6 × 10^−5^, respectively; repeated-measure ANOVA with Tukey's post hoc test; Fig. 3*h*) and increase in power both the low and high gamma bands (*p* = 1.2 × 10^−4^ and *p* = 2.3 × 10^−5^, respectively, repeated-measure ANOVA with Tukey's post hoc test; Fig. 3*h*). Together, this difference in pupil–cortical state coupling between spontaneous pupil dilations and LC stimulation-evoked pupil dilations suggests the possible involvement of other neuromodulatory systems in moderating the coupling between pupil-linked arousal and cortical state.

### CNN classifier analysis confirmed distinct power dynamics across the EEG bands during LC stimulation-evoked and spontaneous pupil dilations

**Figure 4.**
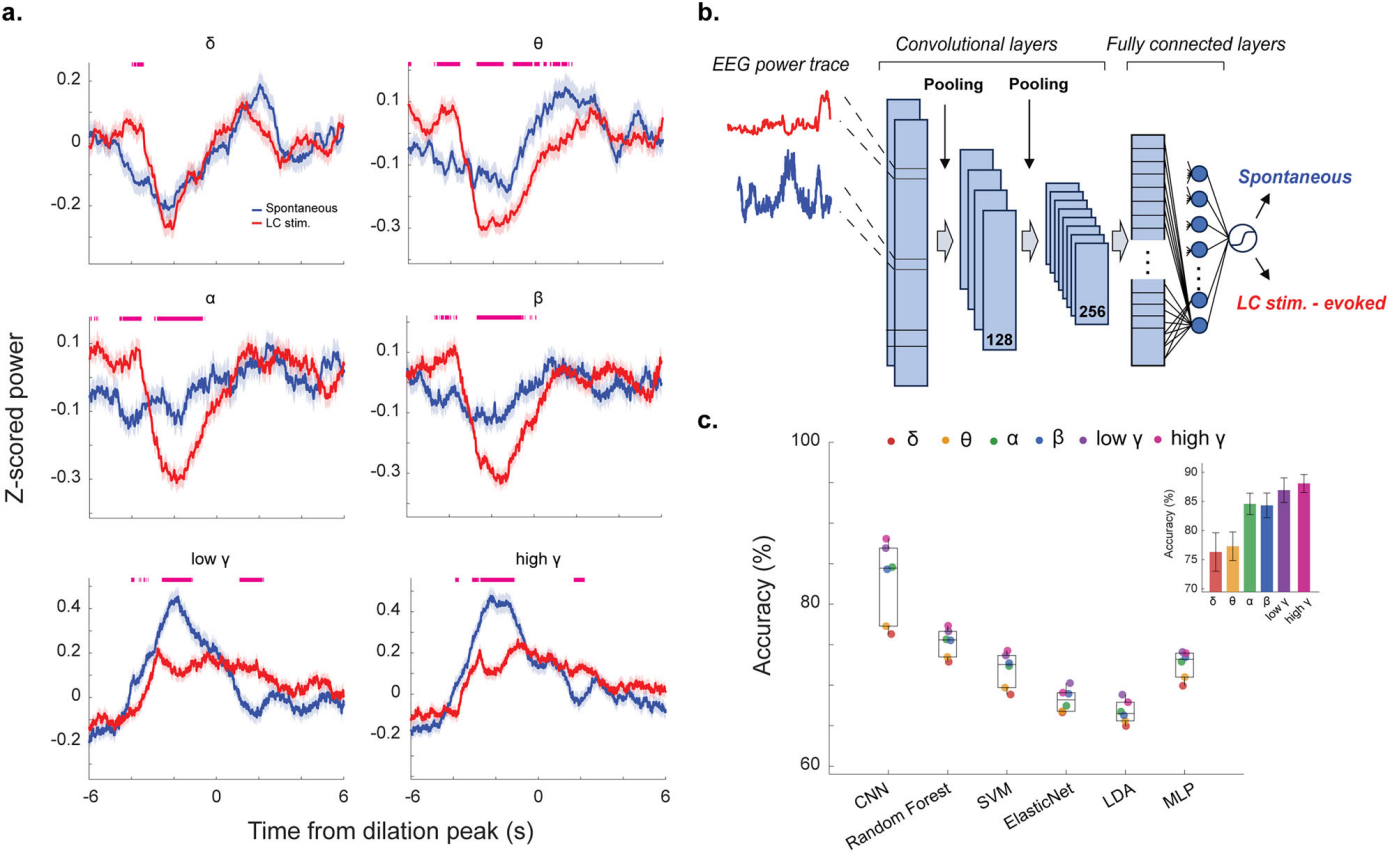
CNN classification of the type of pupil dilations from EEG power dynamics. ***a***, EEG power dynamics of each EEG frequency band around spontaneous and LC stimulation-evoked phasic pupil dilations. Purple marks above *x*-axis indicate statistically significant time points. ***b***, Architecture of the CNN. EEG power traces were fed into a CNN with multiple convolutional, fully connected, and pooling layers with sigmodal output being the network's estimation of a specific dilation being spontaneously evoked or from LC stimulation. ***c***, Model classification accuracy across training folds for each frequency band across all machine learning model types (CNN, random forest, SVM, Elastic Net, LDA, MLP) for 0.5 SD for spontaneous pupil dilation threshold. Inset, CNN accuracy across training folds for each frequency band.

Given spontaneous pupil dilations and LC stimulation-evoked pupil dilations are associated with different EEG power spectra, we looked to further quantify the temporal dynamics of pupil–cortical state coupling. To visualize EEG spectral power variations around phasic pupil dilation periods, we computed PSD using a moving window. This offered higher resolution into band-specific power dynamics over time, which were not measurable with the prior dilation windowing approach (Fig. 4*a*). In this method, EEG power during both LC stimulation-evoked and spontaneous pupil dilations exhibited clear drops in low-frequency (delta and theta) power as well as increases in high-frequency (low and high gamma) power. A point-wise *t* test comparison of each set of power traces confirmed a significant difference between the two conditions in specific periods of all bands: delta (52 significant time points, 2.6% of time series), theta (506 significant time points, 25.3% of time series), alpha (351 significant time points, 17.6% of time series), beta (337 significant time points, 16.9% of time series), low gamma (317 significant time points, 15.8% of time series), and high gamma (366 significant time points, 18.3% of time series). The difference between the traces confirmed the relationship described earlier, where both types of dilations were associated with the shift of cortical state (Fig. 4*a*).

In order to further elucidate the relationship between pupil-linked arousal and cortical state under spontaneous and LC stimulation conditions, we looked to machine learning to help classify the hidden dynamics in each EEG spectral band during LC stimulation-evoked and spontaneous pupil dilations. We developed a CNN classifier to decode if a pupil dilation is from spontaneous elevation of arousal or evoked by LC stimulation using each frequency band's power spectral time series (Fig. 4*b*). In training, the network was fed frequency band-specific EEG power traces associated with the two types of pupil dilations across all animals, passing them through multiple convolutional layers to generate a single sigmoidal output (see Materials and Methods). Our CNN decoder was evaluated using fivefold cross-validation, giving us an average classification accuracy to predict the type of pupil dilation from each EEG power band (Fig. 4*c*). Comparing our CNN results with a wide variety of both supervised and unsupervised machine learning techniques, including random forest, SVM, LDA, as well as Elastic Net and MLP, confirmed the best performance of the CNN for our dataset (Fig. 4*d*). Our CNN classification performance outperformed all other model types, with each band achieving an accuracy of delta (76.77%), theta (77.25%), alpha (84.87%), beta (84.11%), low gamma (86.92%), and high gamma (88.08%), confirming high gamma band to be the most separable between the two types of dilations. Spontaneous dilations detected with thresholds of 1 SD or 0.65 SD yielded similar results, confirming that the dynamics of EEG power associated with spontaneous pupil-linked arousal differ from those evoked by LC activation, most notably in the higher frequencies (Fig. S4).

### Noradrenergic manipulation alters spontaneous pupil and EEG dynamics

**Figure 5.**
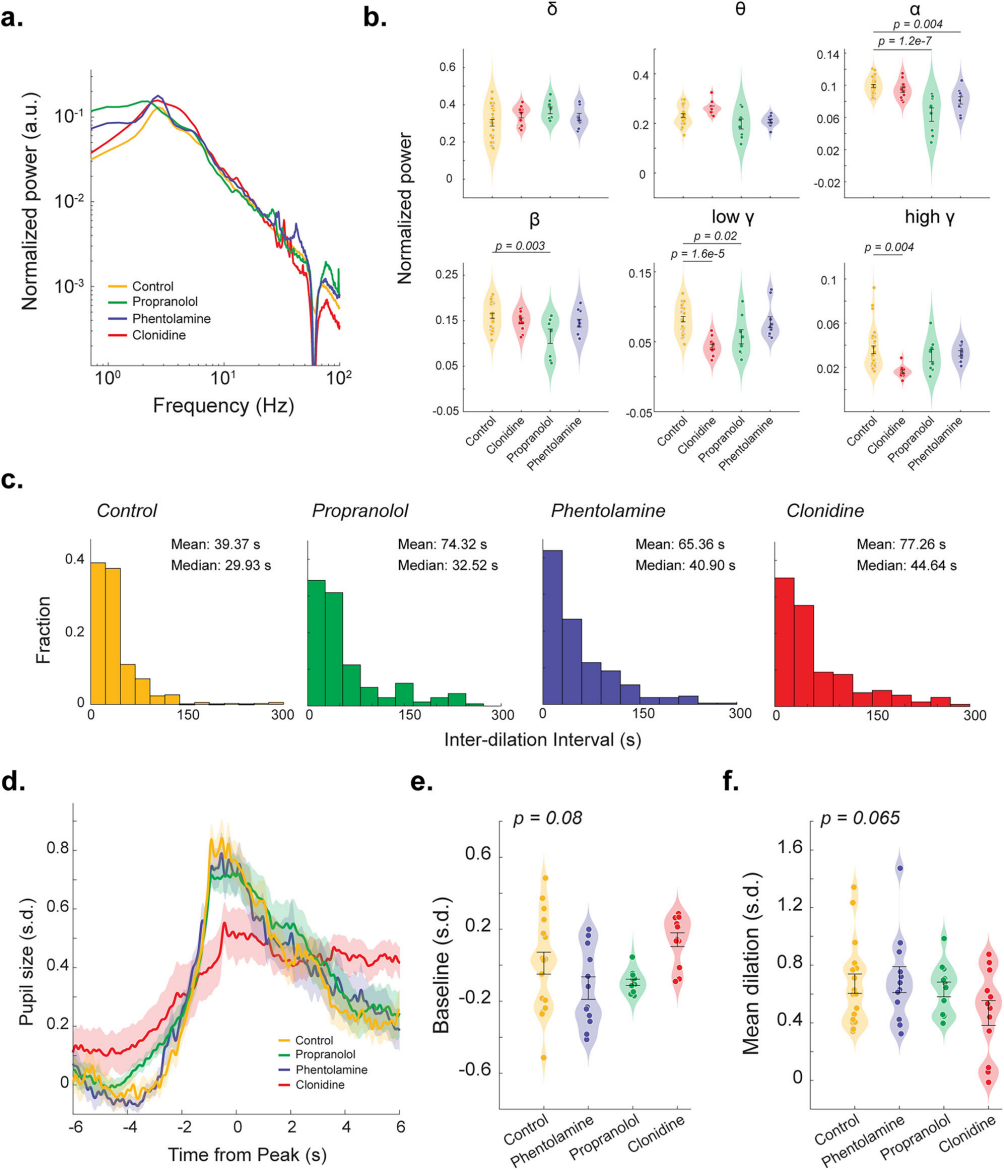
Noradrenergic manipulation alters spontaneous pupil dilations and EEG spectrum. ***a***, Normalized EEG PSD across randomly sampled 2 s periods of activity of all noradrenergic manipulation sessions. ***b***, Normalized EEG spectral power across each frequency band for all manipulation groups. ***c***, Histograms of interdilation intervals for spontaneous phasic pupil dilations. ***d***, Spontaneous pupil dilation across each manipulation and control group, aligned to peak pupil dilation. ***e, f***, The baseline pupil size and mean pupil dilation for each manipulation and control group.

In order to further assess the role of the LC–NE system in moderating the coupling between pupil-linked arousal and cortical state, we evaluated the effects of three FDA-approved adrenergic medications, i.e., clonidine (α-2 agonist), propranolol (β antagonist), and phentolamine (α antagonist), on EEG signals, pupil dilations, and pupil–cortical state coupling. To understand how each drug modulated cortical EEG, we first visualized the EEG power spectral changes across pharmacological manipulation and control sessions (Fig. 5*a*). All three pharmacological manipulations increased EEG power across all bands, most notably in clonidine, implying increased oscillatory activity (Fig. S5*a*,*b*). When assessing the distribution of EEG power over the frequency bands (randomly sampled 2 s periods of activity), we noticed propranolol induced significant decreases in alpha (*p* = 1.25 × 10^−7^, one-way ANOVA post hoc Tukey’s test; Fig. 5*b*), beta (*p* = 0.003, one-way ANOVA post hoc Tukey’s test; Fig. 5*b*), and low gamma (*p* = 0.02, one-way ANOVA post hoc Tukey’s test; Fig. 5*b*) power. Phentolamine significantly decreased alpha (*p* = 0.004, one-way ANOVA post hoc Tukey’s test; Fig. 5*b*) power, while clonidine resulted in significant decreases in low gamma (*p* = 0.02, one-way ANOVA post hoc Tukey’s test; Fig. 5*b*) and high gamma power (*p* = 0.004, one-way ANOVA post hoc Tukey’s test; Fig. 5*b*). Furthermore, we looked to characterize the frequency of spontaneous phasic pupil dilations across sessions for each adrenergic receptor manipulation group (Fig. 5*c*). Interestingly, when compared with the control session's mean interdilation interval (39.37 s), all manipulations slowed the occurrence of spontaneous phasic pupil dilations with propranolol (74.32 s), clonidine (65.36 s), and phentolamine (77.26 s) decreasing the frequency of phasic arousal. We next compared spontaneous phasic pupil dilations between control and manipulation sessions (Fig. 5*d*). Although clonidine group exhibited slightly larger baseline pupil size and reduced pupil dilation, the one-way ANOVA test revealed that there are no statistically significant differences across the four groups (*p* = 0.08, one-way ANOVA for baseline pupil size; *p* = 0.065, one-way ANOVA for pupil dilation; Fig. 5*e*).

Furthermore, to understand the differences in aperiodic components of EEG between each drug manipulation group, we once again performed aperiodic analysis using FOOOF to quantify the slope and offset of EEG spectrum under each drug manipulation. The aperiodic fitting revealed consistent higher aperiodic exponent (*p* = 8.8 × 10^−8^, Student's *t* test) and offset (*p* = 4.8 × 10^−8^, Student's *t* test) for EEG signals under clonidine treatment than control conditions (Fig. S5*c*,*d*). However, there are no significant differences in either aperiodic exponent or aperiodic offset between control conditions and the other two drug manipulations (Fig. S5*c*,*d*).

### Noradrenergic manipulation affects pupil–cortical state coupling during both spontaneous and LC stimulation-evoked phasic pupil dilation

**Figure 6.**
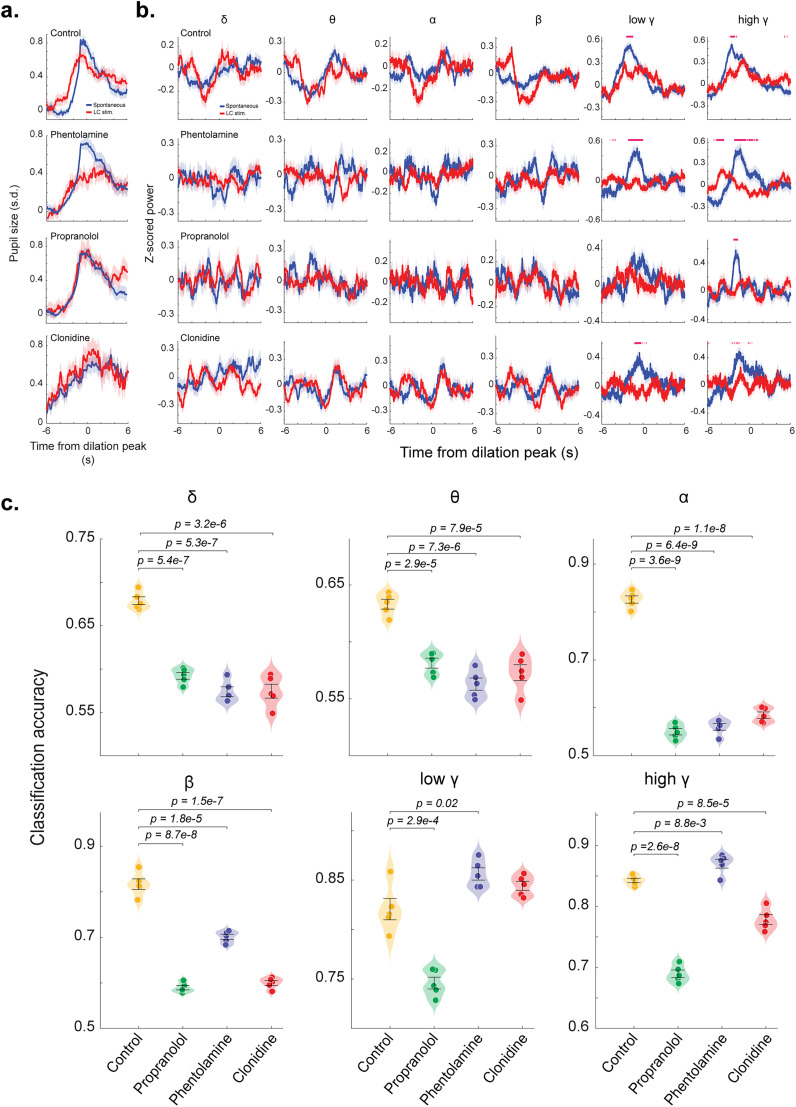
Noradrenergic manipulation reduced the accuracy of the CNN classifier in distinguishing LC stimulation evoked from spontaneous pupil dilations based on EEG signals. ***a***, Spontaneous and LC stimulation-evoked pupil dilations for the control group and each noradrenergic manipulation group. ***b***, EEG power dynamics during spontaneous and LC stimulation-evoked pupil dilations for the control group and each manipulation group. ***c***, Accuracy of the CNN classifier in distinguishing LC stimulation-evoked from spontaneous pupil dilations based on power dynamics of each EEG frequency band.

After comparing spontaneous phasic pupil dilations under different manipulations of adrenergic receptors, we examined the extent to which these pharmacological manipulations affect pupil dilations evoked by LC stimulation. LC stimulation induced smaller pupil dilations in the presence of all pharmacological manipulations compared with control sessions (Fig. 6*a*). Moreover, we observed significant disruptions in low-frequency EEG power dynamics by the manipulations. During sessions with the pharmacological manipulations, we found that dynamics of EEG power in delta, theta, alpha, and beta bands were barely statistically separable between spontaneous and LC stimulation-evoked pupil dilations (Fig. 6*b*). In addition, power in the low and high gamma bands appeared to be not affected by phentolamine during spontaneous pupil dilation, whereas power in both bands decreased during LC stimulation-evoked pupil dilation compared with control sessions (Fig. 6*b*).

To quantify differences in EEG features associated with spontaneous and LC stimulation-evoked pupil dilation under these pharmacological manipulations, we again employed a CNN classifier to classify the type of pupil dilations from EEG power dynamics. This approach mirrored the CNN design used previously, ensuring a consistent spontaneous dilation threshold (0.5 SD) and equal numbers of data for each group were used for classification. As expected, the CNN classifier using high gamma power achieved comparable accuracy under phentolamine manipulation and control conditions. Additionally, both phentolamine and clonidine outperformed the control group in the low gamma band. Interestingly, the classifier using alpha, beta, low gamma, or high gamma power exhibited the lowest accuracy during propranolol manipulation, compared with both phentolamine and clonidine conditions (Fig. 6*c*). Taken together, these results indicate that all three subtypes of adrenergic receptors play a critical role in modulating the coupling between pupil-linked arousal and cortical state.

## Discussion

In the present study, we investigated the extent to which the LC–NE system contributes to pupil-linked arousal coupling to cortical state. This question is grounded in the critical role of the LC–NE system in regulating brain-wide arousal levels and influencing neural information processing (Berridge and Waterhouse, 2003; Eschenko et al., 2012; Rodenkirch et al., 2019; Rodenkirch et al., 2022; Glennon et al., 2023; Ghosh and Maunsell, 2024). Pupil diameter has become a valuable, noninvasive readout of internal arousal state and, more specifically, is tightly correlated with LC–NE activity (Joshi et al., 2016; Reimer et al., 2016). While prior literature has provided functional evidence that direct activation of the LC can evoke both pupil dilations and concordant EEG power changes (typically reflecting cortical desynchronization; Berridge and Foote, 1991; Carter et al., 2010), similar fluctuations in the pupil size and cortical state also occur spontaneously (Yoss et al., 1970; Reimer et al., 2014, 2016; McGinley et al., 2015; Pfeffer et al., 2022). However, a direct comparison quantifying how the relationship between cortical state during direct LC-evoked and spontaneous arousal fluctuations has remained unexplored. Our study provides new evidence supporting a difference in cortical state during spontaneous phasic arousal and LC stimulation-evoked phasic arousal. Moreover, our pharmacological manipulations of adrenergic receptors reveal the importance of each subtype of adrenergic receptors in moderating cortical oscillations and their coupling to the pupil size, suggesting nuances in how NE release from phasic LC activation impacts cortical dynamics compared with endogenous arousal shifts.

A critical aspect of our study is the comparison between pupil dilations evoked by direct LC phasic stimulation versus those arising spontaneously, as these events likely engage distinct underlying neural circuits and neuromodulatory contexts, potentially leading to differential coupling with cortical activity. Pupil dilations evoked by LC activation are primarily driven by a rapid and phasic release of NE that influences autonomic control pathways, including projections involving the Edinger–Westphal nucleus (EWN) complex (Breen et al., 1983; Samuels and Szabadi, 2008; Liu et al., 2017). Direct optogenetic stimulation of the LC bypasses the complex integration of afferent input from broader brainstem nuclei that normally shape LC firing. It is important to note that while optogenetic stimulation reliably activates the LC, its effects may influence the activity of other neuromodulatory systems like the basal forebrain (Beani et al., 1978; Bianchi et al., 1979; Liu et al., 2025). However, it is plausible that the changes in EEG and the pupil size following LC stimulation primarily resulted from the activation of the LC–NE system. In contrast, spontaneous phasic pupil-linked arousal may arise from a distributed neuromodulatory network, likely with inputs from other regions such as the prefrontal cortex, hypothalamus, and superior colliculus (Wang and Munoz, 2015; Schneider et al., 2016). This network collectively regulates the activity of the autonomic and neuromodulatory systems, including the LC itself, often resulting in larger pupil dilations and reflecting complex, ongoing cognitive and arousal states. In our study, we observed that during phasic arousal—either occurred spontaneously or evoked by LC stimulation—EEG oscillatory power shifted from low-frequency bands to higher-frequency bands, a transition indicative of a rapid cortical sate shift associated with heightened arousal (Carter et al., 2010; Sara and Bouret, 2012). However, the context for the EEG power shifts differs: spontaneous phasic pupil-linked arousal may involve the contribution of additional neuromodulatory inputs. Serotonergic neurons from the dorsal raphe nucleus, whose phasic activity has been shown to influence the pupil size (Cazettes et al., 2021; Maheu et al., 2025), modulate arousal and autonomic tone, potentially acting independently or synergistically with the LC–NE system (Pickel et al., 1977; Szabo and Blier, 2001; Maheu et al., 2025). Similarly, cholinergic input via acetylcholine from the basal forebrain is crucial for regulating cortical excitability and driving transitions between synchronized and desynchronized cortical states (Goard and Dan, 2009; Pinto et al., 2013; Lin et al., 2015; Xu et al., 2015). Notably, cortical cholinergic activity has been shown to correlate with fluctuation of the pupil size (Nelson and Mooney, 2016; Reimer et al., 2016; Liu et al., 2025). Therefore, while the sequence of cortical activation preceding pupil dilation appears generalizable, the distinct underlying circuitry and broader neuromodulatory context (including NE, 5-HT, ACh, and potentially others) associated with spontaneous versus LC stimulation-evoked phasic arousal likely contributes to the specific differences in pupil–cortical state coupling revealed in our study. Future work investigating these differences could elucidate the precise contribution of each neuromodulatory signaling to cortical arousal state relative to more globally integrated arousal processes.

NE exerts complex modulatory control over brain functions via distinct adrenergic receptor subtypes (α-1, α-2, and β), each coupled to different intracellular signaling pathways and exhibiting unique distributions (Ramos and Arnsten, 2007; O’Donnell et al., 2012; Slater et al., 2022). To dissect the contribution of α-adrenergic receptors to NE's influence on cortical state, we employed phentolamine, a nonselective α antagonist (Rodenkirch et al., 2019). This administration seemingly disrupted EEG oscillations in the delta, theta, alpha, and beta bands during both spontaneous and LC stimulation-evoked pupil dilation periods. However, power in both low and high gamma bands was not affected during spontaneous pupil dilations, whereas it was disrupted during LC stimulation-evoked pupil dilations. While consistent with previous studies showing that α-adrenergic receptors mediate NE's influence on cortical state (Berridge and Waterhouse, 2003; Berridge and Spencer, 2016; Ma et al., 2024), our findings provide new evidence that α-adrenergic receptor signaling constitutes a necessary pathway specifically linking phasic NE release to the modulation of cortical circuits underpinning slow oscillations. The clear distinction between gamma power during LC stimulation-evoked pupil dilations and spontaneous pupil dilations suggests that neurotransmitters other than NE may contribute more significantly to gamma oscillations (Fig. 4*a*).

Administration of clonidine, an α-2–adrenergic agonist, resulted in a reduction of gamma power during spontaneous phasic arousal. α-2–adrenergic receptors are densely expressed in LC neurons and their terminals (Marwaha and Aghajanian, 1982; De Sarro et al., 1987; Chamba et al., 1991). Activation of these Gi GPCRs hyperpolarizes LC neurons and suppresses NE release, exerting powerful control over central noradrenergic tone and, consequently, arousal and cortical network activity (Hein et al., 1999; Berridge and Waterhouse, 2003). Moreover, the expression of α-2–adrenergic receptors in the EWN mediates the control of the pupil size by the LC in the parasympathetic pathway (Liu et al., 2017). Consistent with clonidine's reported effects, we observed changes in pupil dynamics linked to spontaneous phasic arousal and LC stimulation, including larger baseline pupil size and longer interdilation intervals (Gherezghiher and Koss, 1979; Koss and Christensen, 1979; Hou et al., 2005). The inhibition of the EWN by clonidine slowed down phasic pupil dilation evoked by LC stimulation (Fig. 6*a*). There is also substantial evidence of the LC's activation also inhibiting cortical ACh release, potentially through the activation of presynaptic α-2 receptors in the basal forebrain and on cortical cholinergic nerve endings (Beani et al., 1978; Bianchi et al., 1979; Acquas et al., 1998). Additionally, clonidine has been linked to directly block muscarinic cholinergic receptors, implicating that its effects may be less specific in nature to a true α-2 agonism (Buccafusco, 1982; Buccafusco and Aronstam, 1986). Moreover, clonidine resulted in a disruption in the coupling between spontaneous pupil-linked arousal and low-frequency EEG activity. This aligns with literature showing a reduced noradrenergic tone via α-2 agonism, leading to increased cortical synchrony and therefore low-frequency power (Florio et al., 1975; Pastel and Fernstrom, 1984; Riekkinen et al., 1990). Our results extend this finding by demonstrating that the dampening of the NE system effectively eliminates the relationship between phasic NE release and low-frequency cortical EEG activity. This decoupling could take effect by suppressing presynaptic NE to levels insufficient to reliably drive or coordinate downstream low-frequency cortical network activity.

Another interesting finding is that the manipulations of α-adrenergic receptors mostly disrupted EEG power during spontaneous phasic pupil dilations in the delta, theta, alpha, and beta bands, but not in gamma bands (Fig. 6*a*). This suggests that the central pupil-linked arousal system modulates gamma oscillations not through alpha adrenergic receptors. However, when blocking β-adrenergic receptors, EEG power across all bands was disrupted. As G-protein–coupled receptors, β-adrenergic receptors activate adenylyl cyclase and downstream cAMP/PKA signaling pathways (Molinoff, 1984; Johnson, 1998). This intracellular cascade is well known to enhance neuronal responsiveness, facilitate synaptic plasticity such as long-term potentiation (Gelinas and Nguyen, 2005; Zhang et al., 2013; Hagena et al., 2016; Borodovitsyna et al., 2017), and contribute to sustained arousal and attentional processes (Pattij et al., 2012; André et al., 2015). Furthermore, β-adrenergic receptors are implicated in neural desynchronization and affect high-frequency oscillations, which are thought to reflect improved cortical processing and information transmission (Roubicek, 1976; Hazra et al., 2012). The broad disruption of EEG power by propranolol administration, particularly in high frequencies, aligns with the known effect of propranolol on high-frequency network activity (Orzack et al., 1973; Haggerty et al., 2013). The impact of β-adrenergic receptor inhibition across all frequency bands, contrasting with α-adrenergic receptor inhibition, might reflect the widespread distribution of β-adrenergic receptors throughout brain structures (Wanaka et al., 1989; Cox et al., 2008) or the downstream signaling cascades which can broadly amplify NE's modulatory influence on overall network excitability (Waterhouse et al., 1982). Taken together, our results highlighted the critical role of adrenergic receptors in maintaining the NE-driven coordination of cortical networks that underlies robust pupil–cortical coupling during arousal events. Future work is warranted to test the role of other neurotransmitter receptors, including nicotinic and muscarinic cholinergic receptors, dopaminergic receptors, and serotoninergic receptors, in moderating the coupling between pupil-linked arousal and cortical state.
